# Healthcare worker perceptions of the FAST TB infection control strategy

**DOI:** 10.5588/pha.24.0055

**Published:** 2025-06-04

**Authors:** D.B. Tierney, R.R. Nathavitharana, L. Cummins, K. Tintaya, A. Biewer, R. Guerrero, L. Lecca, L.R. Hirschhorn, E.A. Nardell, A.K. Nelson

**Affiliations:** ^1^ Brigham and Women’s Hospital, Boston, MA, USA;; ^2^ Harvard Medical School, Boston, Massachusetts, USA;; ^3^ Beth Israel Deaconess Medical Center, Boston, MA, USA;; ^4^ Duke University Medical School, Durham, NC, USA;; ^5^ Socios En Salud, Lima, Peru;; ^6^ Hospital Nacional Hipolito Unanue, Lima, Peru;; ^7^ Northwestern University Feinberg School of Medicine, Chicago, IL, USA;; ^8^ Yale School of Public Health, Department of Health Policy and Management, New Haven, CT, USA.

**Keywords:** tuberculosis, Peru, qualitative research, care cascade

## Abstract

**BACKGROUND:**

The FAST (Find cases Actively, Separate safely and Treat effectively) TB infection control (TB-IC) strategy decreases the time-to-TB diagnosis and treatment. We examined healthcare workers’ (HCW) perceptions of FAST implementation at a tertiary referral hospital in Lima, Peru.

**METHODS:**

From August 2016 to December 2019, we conducted 24 interviews and four focus groups (*n* = 14) with a diverse population of HCWs and other stakeholders involved throughout the TB care cascade. We used inductive analysis to identify emergent themes, used Dedoose to code transcripts accordingly, and developed a narrative using reflexive thematic analysis.

**RESULTS:**

We identified three emergent themes: 1) FAST impact on TB care, 2) FAST impact on TB-IC and 3) FAST impact on hospital culture. FAST was felt to improve the speed of TB screening, diagnosis and treatment. TB specialists recognized that by expediting diagnosis and treatment, FAST likely decreased transmission. Healthcare workers appreciated that FAST improved care coordination along the TB care cascade. FAST changed hospital culture related to the prioritization of TB-IC.

**CONCLUSION:**

HCWs perceived that FAST, implemented by a dedicated external team, was an acceptable and effective TB-IC strategy that improved care coordination and overall TB quality of care.

TB transmission is an important concern in hospitals in TB-endemic countries.^1^ Transmission is driven by patients with untreated TB, either because TB is unsuspected and undiagnosed or because of delays in initiating effective treatment, especially in those with drug-resistant TB.^2,3^ The TB infection control (TB-IC) strategy known as FAST (Find cases Actively, Separate safely and Treat effectively) was developed to decrease TB transmission in hospitals by rapidly identifying and treating patients with TB at the outset of hospitalization.^2^ FAST functions on two principles: 1) universal screening for TB disease, followed by prompt diagnosis and drug susceptibility testing (DST); and 2) effective TB treatment to rapidly render TB disease non-infectious.^4^ Thus, the FAST TB-IC strategy improves the quality of TB care and, as a consequence, decreases TB transmission.^5–7^ There is emerging evidence that FAST is an effective TB-IC intervention. In a Bangladesh chest hospital, FAST detected a high frequency of unsuspected TB, particularly in patients with chronic lung diseases who had a prior history of TB.^8^ FAST using Xpert^®^ MTB/RIF (Cepheid, Sunnyvale, CA, USA) as the initial diagnostic test increased the proportion of patients initiating likely effective TB treatment within two days of diagnosis at a TB hospital in Vietnam.^9^ FAST also decreased the time to detection, diagnosis, and treatment of patients with multidrug-resistant (MDR)-TB in Nigeria.^10^ FAST implementation at a general tertiary hospital in Peru increased the likelihood and speed of pulmonary TB treatment initiation and the likelihood of MDR-TB diagnosis.^7^ FAST has also been associated with a 78% reduction in the risk of acquiring MDR-TB for patients hospitalized at two hospitals in Russia.^11^

Guidance from international health organizations has long characterized administrative TB-IC interventions that include active case-finding strategies like FAST as among the most effective.^12,13^ Guidance on how best to implement these strategies, however, has been limited. They are thus not widely used.^14,15^ Health system factors are prominent implementation barriers, including limited material and human resources to support TB-IC.^15,16^ Healthcare workers’ (HCW) perceptions of TB-IC practices also have an important influence on implementation.^17,18^ To understand the process and impact of FAST implementation by a dedicated team as part of a prospective intervention trial at a tertiary referral hospital in Lima, Peru, we conducted a qualitative study to explore HCW perceptions of FAST for its 1) impact on patient care 2) effectiveness as an administrative TB-IC strategy and 3) implementation barriers and enablers.

## METHODS/DESIGN

### Study setting

Peru has a moderate burden of TB, with a total incidence of 151/100,000 population.^19^ Hospital Nacional Hipólito Unanue (HNHU) is a 700-bed public general hospital in Lima, Peru, which cares for approximately 250 patients with newly diagnosed TB annually. The FAST strategy was implemented at HNHU from August 2016 to December 2019. FAST activities were primarily performed by a team of implementers from Socios en Salud (SES), a Peruvian non-governmental organization. Activities were integrated into the hospital’s existing TB protocols, as shown in the Figure, with FAST implementers coordinating with HNHU staff across the TB care cascade. Upon entering the hospital, patients were screened for cough and other TB risk factors and offered enrolment in the FAST study if screening was positive.^7^ Patient sputum samples were collected and tested for TB with smear microscopy and line probe assay—the HNHU standard protocol—until August 17, 2017, when the FAST study introduced Xpert as an additional diagnostic test. FAST implementers communicated test results from the laboratory to hospital clinicians, who were responsible for initiating TB treatment. Representatives from the Peruvian National Tuberculosis Program (NTP) based at HNHU logged the patient in the national TB registry and arranged for follow-up outpatient management upon discharge.

**FIGURE.**
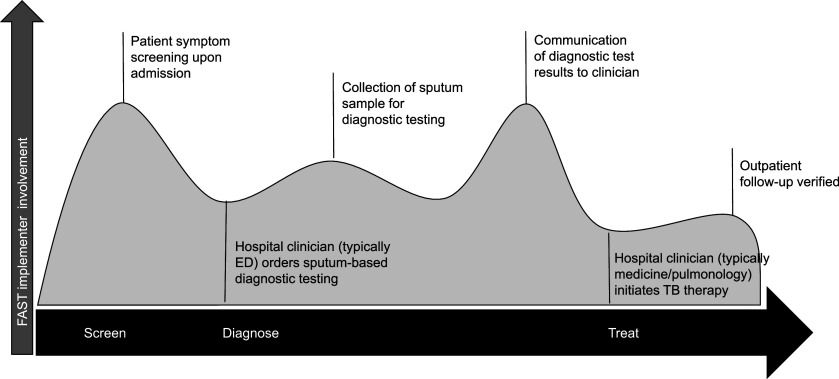
Intensity of FAST implementer involvement throughout the hospital TB care cascade at HNHU. FAST = Find cases Actively, Separate safely, Treat effectively; HNHU = Hospital Nacional Hipolito Unanue; ED = Emergency Department.

### Study population and data collection

Study reporting aligns with the Consolidated criteria for reporting qualitative research (COREQ) (Supplementary Data).^20^ SES personnel trained in qualitative research conducted interviews and focus groups with HNHU staff and FAST implementers about their experience with FAST. A semi-structured interview guide was used to assess the HCWs’ roles and work tasks related to FAST and their perceptions of the strategy. Interviews were conducted, recorded, and transcribed in Spanish, then translated into English by LC, a fluent Spanish speaker. Twenty-four interviews and four focus groups (30–60 min) were conducted between 2016 and 2019, allowing longitudinal assessment and data saturation regarding perceptions about FAST during the 4-year implementation period (Table 1). Interview participants were identified by the SES implementation team using convenience sampling from three cohorts with different roles in the TB care cascade to understand perceptions about FAST as a function of work tasks. ‘ED clinicians’ play a key role at the front line of TB screening and diagnosis. The “TB care specialist” cohort was selected for their specific involvement with TB patient care at HNHU, including laboratory testing for TB, clinical management of respiratory diseases including TB, or programmatic TB control. The “FAST implementers” cohort comprised SES staff on the team implementing FAST at HNHU. Focus groups were primarily conducted with the FAST implementation team and ED nursing assistants, who were more easily able to participate as part of a group than other HCWs.

**TABLE 1.** List of participants and roles for interviews and focus groups conducted at Hospital Nacional Hipolito Unanue, 2016–2019.*

2016	2017	2018	2019
ED Nurse Director (1) ED Physician A (1) Head of Research (1) Infectious Disease Nurse • NTP Nurse A • Hospital Epidemiologist (1) • Head of Emergency and Critical Care • Chief Pulmonologist (1)	• ED Nurse Director (2) • Head NTP Nurse • ED Physician A (2) • Head of Research (2) • Laboratory Coordinator • Hospital Epidemiologist (2) • Chief Pulmonologist (2)	• ED Nurse A ED Nurse B • ED Nurse C • ED Nurse D • ED Nursing Assistant • ED Physician B	• NTP Nurse B • NTP Nurse C • Chief Pulmonologist (3)
	• Focus Group (8 participants): FAST implementers (1) • Focus Group (8 participants): FAST implementers (2)	• Focus Group (6 participants): ED Nursing Assistants	• Focus Group (8 participants): FAST implementers (3)

* Number in parentheses represents interview or focus group number in series. Letter following title of HCW is used to denote different individual participants within a HCW cadre.

ED = Emergency Department; NTP = National TB Program; HCW = healthcare worker.

### Data analysis

Reflexive thematic analysis was used to analyze data.^21^ First, three study team members (DT, LC, and AKN) read interview transcripts and generated a list of themes. The codebook was created based on these emergent themes and study objectives. LC and AKN double-coded, compared and discussed five interviews to fine-tune the codebook. Interview transcripts and the finalized codebook were uploaded into Dedoose 8.1 (SocioCultural Research Consultants, LLC., Los Angeles, CA). Inter-coder reliability was assessed, and double coding was repeated until an 80% agreement between coders was reached. LC then coded the remaining 19 interviews and four focus groups in Dedoose. Themes were identified from paragraph summaries written by DT, LC, and AKN, and a narrative connecting these themes emerged from discussions with study team members, including KT and RN.

IRB approval was obtained from the ethics board at HNHU and Brigham and Women’s Hospital. All participants provided written informed consent.

## RESULTS

We identified three emergent themes from interview and focus group transcripts: 1) FAST impact on TB care, 2) FAST impact on TB infection control and 3) FAST impact on hospital culture. Illustrative quotations are detailed in Table 2.

**TABLE 2.** Illustrative quotations organized by themes.

Theme	Participant quotation
FAST impact on TB care	
	“[FAST] does help, through active case finding... I oversee the identification of patients with respiratory symptoms. The FAST team is very helpful because they do help to identify patients.”
	NTP nurse B, 2019
	“Before your [FAST] team started we did smear microscopy tests but they were delayed. The lab would tell us that a result should come out in four hours, but it wasn’t true; the result came out after ten or twelve hours and you… didn’t know if [the patient] was negative or positive. With your [FAST] team, this process is getting faster.”
	Head ED nurse, 2016
	“…The results aren’t getting to [the clinicians]. Since [FAST implementers] have been here, this has gotten much better. You have a team that is waiting for the results…to add them to the medical chart and let the doctor know.”
	Laboratory coordinator, 2017
	“I think that there are many benefits because if they are diagnosed, they stop transmitting TB. And if I also get their sensitivity test I’m going to give them an appropriate treatment right for the patient.”
	Chief pulmonologist, 2019
	“I like [FAST] because we are helping patients—finding new cases and patients with drug resistance… to ensure that they receive better treatment.”
	FAST technician, 2017
	“What I understand is how much FAST helps us with the normal care we provide.”
	ED nursing assistant, 2018
FAST impact on TB infection control	
	...In general, it’s risky in the hospital. The entire hospital is a major [TB transmission] risk area, of course, because we care for patients who have known [TB] disease on arrival. But generally, on the wards for those patients, there is some attention paid to managing infection control. The real danger is in places where we don’t suspect we have a patient [with TB] as a user of our services.
	Chief pulmonologist, 2016
	“We don’t have enough of a commitment [to TB-IC] by the hospital, knowing that we’re working in a high-risk area. Not having adequate infection control measures seems to me like playing Russian roulette with our own health. It’s evident that if we can find patients [with TB] more quickly, it will be much better.”
	ED physician A, 2016
	“...for patients that come to the hospital with problems that aren’t necessarily related to tuberculosis, they have a TB test and it comes out positive, so it makes you alert. That you should protect yourself more, right? That all patients are potentially infectious.”
	FAST technician, 2017
	“It is important that [TB] diagnosis, follow-up and immediate treatment are done to reduce the risk [of transmission]. If we do not make a timely diagnosis, this patient may spend one or two or three days in the emergency department while smear positive, where there is a high risk of contagion for the staff. On the other hand, if we diagnose TB and start the treatment, the risk of transmission to staff goes down.”
	NTP nurse A, 2016
	“FAST personnel enter a ward wearing a respirator properly. The worker who normally works in that ward then sees how a person should protect himself or herself—how to [wear a respirator], they see the way to do it. So, say I work there on this ward, and I don't feel like it [wearing a respirator] or I don't have the right respirator, so I don't protect myself, but then I see that a healthcare worker coming in is using the protective measures properly. I can learn from that person how they take care of themselves.”
	Chief pulmonologist, 2016
FAST impact on hospital culture	
	“When it comes to handing in the [sputum test] result, if it is positive, it is honestly like a diploma. You go and say to the nurse, ‘the patient is positive’ and they finally pay attention to you... It is that lack of interest of some of the staff that creates an obstacle and slows everything down. For me, it holds things up to not have the patient chart, for example, or not be able to request the chart.
	FAST technician, 2017
	“We have patients who have been in the hallways for three, four days and if you don’t move them to pulmonology [ward]… they will stay in the hallways, no? So we need that—better coordination with [pulmonology]. We are exposed to illness here, as staff in the emergency room, because there is not fast enough movement.”
	ED nurse director, 2016
	“In terms of my staff, they are now all aware [of the need for coordinated care] and they even go look for you [FAST implementers]. (Laughs.) They go looking for you. The residents ask me, ‘Where are the [FAST] ladies?’ (Laughs.) ‘They are probably in their office’, I say. They go looking for you. They are aware and that is good because you knew how to reach them.”
	ED nurse director, 2016
	“We put together a new TB control plan, where several aspects of FAST are considered, especially the quick diagnosis has been incorporated into the plan, thanks to scientific evidence presented by FAST.”
	Chief pulmonologist, 2017

FAST = Find cases Actively, Separate safely, Treat effectively; NTP = National TB Program; ED = Emergency Department.

### FAST impact on TB care

Most participants reported that FAST had benefits throughout the care cascade, from screening to diagnosis to treatment, improving the hospital’s standard of care. Observations were consistent with HCWs’ clinical roles and reflected their work. For example, participants responsible for screening noted the utility of FAST in increasing the capacity to screen a larger population of patients with respiratory symptoms. ED clinicians, who are primarily responsible for evaluating patients with suspected TB, perceived that FAST increased the number of patients diagnosed with TB and emphasized the benefit of Xpert for diagnosis. HCWs mentioned the specific benefit of a dedicated implementation team from SES in improving screening capacity and shortening time-to-diagnosis by speeding diagnostic test results delivery and alleviating bottlenecks due to long laboratory wait times. Laboratory technicians similarly reported that FAST shortened the time to TB diagnosis. In contrast to ED clinicians, the laboratory technicians described diagnostic delays resulting from inefficient care coordination in the ED. Lab technicians attributed TB improved care coordination to FAST presence at the hospital. Pulmonologists overseeing TB treatment initiation recognized the benefits of using Xpert, which provides rapid first-line DST as part of FAST implementation.

In contrast to HCWs, whose comments were specific to a step of the TB care cascade for which they were personally responsible, FAST implementers spoke more holistically about how the principles of FAST could yield patient care benefits.

### FAST impact on TB infection control

Employees at HNHU recognized their risk of contracting TB at work and were realistic about where and how TB was transmitted to HCWs. Frontline ED clinicians praised FAST for increased TB detection. They felt that patients with unsuspected TB who were being cared for in the ED for non-TB illnesses posed a risk to healthcare workers and other patients as potential sources for TB transmission. Multiple participants stressed the need for rapid initiation of TB therapy to decrease TB transmission in the hospital. TB care specialists demonstrated an understanding that treating a patient with a likely effective TB regimen informed by drug susceptibility testing has a high likelihood of decreasing the contagiousness of the patient, especially in those patients with high bacterial burden in respiratory secretions as indicated by positive sputum smear microscopy.

‘FAST as indirect infection control training’ emerged as an important secondary theme. Participants discussed how the presence of the FAST implementation team increased awareness about TB-IC across hospital departments. Participants commented on the FAST implementers’ use of personal protective equipment (PPE), which served as an example of how health workers should prioritize their health and safety. FAST implementers echoed this sentiment, stating that FAST provided a constant reminder to clinicians that any patient could have TB which in turn improved HCW behaviours around TB infection control.

### FAST impact on hospital culture

Our data provided insights into how FAST improved the coordination of TB care by helping to connect the ED, laboratory, and medical teams. We also identified resultant tensions between those providing general frontline patient care and those whose work was more focused explicitly on TB care, often due to resources, including bed constraints. FAST implementers expressed concerns that hospital clinicians had limited insights about how their work fits into the larger TB care cascade due to their focus on day-to-day clinical tasks and that clinicians were sometimes resistant to the additional clinical tasks required by FAST with complaints about extra work. Clinicians, including nurses, highlighted the importance of education about FAST, particularly when considering new staff.

ED clinicians often mention the challenges of implementing TB-IC measures. They expressed feelings of stress around coordinating care with multiple specialists, both for initial referrals and follow-ups. While coordination between clinical services responsible for different aspects of the care cascade was identified as a challenge to high-quality TB care, FAST was generally seen as a positive force to address these challenges. Those in the TB care specialist cohort agreed with FAST implementers and ED nurses about FAST changing hospital culture around TB prevention. One TB care specialist mentioned that the FAST protocol has been incorporated into the hospital’s TB care guidelines

## DISCUSSION

FAST is a complex implementation strategy that relies on the coordinated efforts of various actors, from emergency clinicians to laboratory staff to TB-focused clinicians. Qualitative data from interviews and focus groups at HNHU, a general hospital in Lima, Peru, where FAST was implemented between 2016 and 2019, indicate that clinicians and laboratory staff involved in TB care perceived FAST as an acceptable and effective strategy. HCWs highlighted that FAST improved TB care, primarily through expedited diagnosis and treatment initiation, which aligns with our prior analyses demonstrating that FAST decreases time-to-diagnosis and treatment^7^. HCWs perceived that the FAST implementation team facilitated the flow of clinical information and care coordination in the complex hospital environment, improving patient progress through the TB care cascade.

The approval of FAST and its subsequent integration into TB care guidelines at HNHU likely reflects recognition by HCWs that the intervention appropriately addressed problems widely recognized and frequently encountered in their daily clinical work, which is critical to supporting procedural changes in healthcare settings.^22,23^ Perceptions about the speed of patient progress through the care cascade were also likely influenced by the introduction of Xpert as a new diagnostic test at HNHU as part of an active TB screening process and the benefits of FAST team care coordination. Distinct from TB care quality improvement, FAST was perceived as a valuable TB-IC intervention, a view most prominently held by TB specialists. We found significant concerns about occupational TB risk and a desire for safe workspaces across the HCW groups at HNHU. This aligns with data from other settings.^24^ We hypothesize that FAST was well-received partially because it was recognized as a TB-IC intervention that was perceived to create a safer work environment. FAST implementers, in role-modeling TB-IC activities, seem to motivate other hospital staff to engage in TB-IC, especially in the use of PPE. Improved awareness of the active role HCWs can play as change agents can strengthen TB-IC implementation.^18^ Our study provides additional evidence to suggest that HCW TB-IC activities can be influenced by their peers.

Introducing a strategy like FAST at a large general hospital in a TB-endemic setting within an existing care infrastructure is disruptive. For a patient to successfully progress through the TB care cascade, healthcare workers in administrative, laboratory and clinical roles must perform a series of tightly coordinated tasks focusing on minimizing delays in care.^8^ This complex level of interaction poses challenges, especially for understaffed roles.^15^ The FAST implementation team identified feelings of resentment and friction that sometimes occurred during interactions with HCWs, including blame for patient care delays on other hospital departments. These stresses and resource constraints are common to TB-endemic settings and highlight the need for increased funding for National TB Programs to ensure the resources, including access to molecular testing and PPE for HCWs, that are needed to improve TB care quality.^25^

The type of personnel that are designated to implement FAST may ultimately determine whether implementation is sustainable. In our study, FAST was executed by an additional cadre of workers who offloaded some daily clinical tasks from clinicians. While this implementation approach may support effective future scale-up of intensified case-finding TB-IC strategies like FAST, it may be a threat to sustainability if it cannot be replicated at other sites due to resource constraints. As hospitals consider scaling up administrative TB-IC strategies like FAST, evaluations that compare implementation with existing staff versus a dedicated team of implementers will help determine how FAST can be best operationalized and sustained in high TB incidence settings.

Limitations of our study include convenience sampling used to identify willing and available participants, which could introduce selection bias. Although the study was implemented from 2016 to 2019, few participants were interviewed more than once, aside from the FAST implementation team, which limited our ability to identify how perceptions and attitudes changed over time.

## CONCLUSIONS

Our findings have important implications for the wider implementation and dissemination of the FAST strategy. Given that TB-IC has been historically poorly implemented and depends on HCW buy-in,^26^ marketing FAST as a transmission prevention strategy of value to HCWs and patients may increase uptake in TB-endemic settings. The conceptualization of FAST as a TB care quality improvement intervention could be leveraged to build support among hospital staff caring for patients along the TB care cascade. TB specialists, who are more attuned to the concept of ‘treatment as prevention’^3^ may be motivated by the TB-IC potential of FAST and can serve as champions at FAST implementation sites.
